# Effects of microRNA-135a on the epithelial–mesenchymal transition, migration and invasion of bladder cancer cells by targeting GSK3β through the Wnt/β-catenin signaling pathway

**DOI:** 10.1038/emm.2017.239

**Published:** 2018-01-19

**Authors:** Xia-Wa Mao, Jia-Quan Xiao, Zhong-Yi Li, Yi-Chun Zheng, Nan Zhang

**Affiliations:** 1 Department of Urology Surgery, The Second Affiliated Hospital of Zhejiang University Medical College, Hangzhou, P.R. China

## Abstract

This study investigated the effects of microRNA-135a (miR-135a) targeting of glycogen synthase kinase 3β (GSK3β) on the epithelial–mesenchymal transition (EMT), migration and invasion of bladder cancer (BC) cells by mediating the Wnt/β-catenin signaling pathway. BC and adjacent normal tissues were collected from 165 BC patients. Western blotting and quantitative real-time PCR were used to detect the expression of GSK3β, β-catenin, cyclinD1, E-cadherin, vimentin and miR-135a in BC tissues and cells. Cells were assigned to blank, negative control (NC), miR-135a mimics, miR-135a inhibitors, small interfering RNA (siRNA)-GSK3β or miR-135a inhibitors+siRNA-GSK3β groups. miR-135a, β-catenin, cyclinD1 and vimentin expression increased, while GSK3β and E-cadherin expression decreased in BC tissues compared with adjacent normal tissues. Compared with the blank and NC groups, the expression of miR-135a, β-catenin, cyclinD1 and vimentin was higher, and cell proliferation, migration, invasion and tumor growth were increased in the miR-135a mimics and siRNA-GSK3β groups. These groups showed an opposite trend in GSK3β and E-cadherin expression and cell apoptosis. The miR-135a inhibitors group was inversely correlated with the blank and NC groups. It was concluded that miR-135a accelerates the EMT, invasion and migration of BC cells by activating the Wnt/β-catenin signaling pathway through the downregulation of GSK3β expression.

## Introduction

Bladder cancer (BC) is one of the most prevalent cancers worldwide. BC is a common genitourinary malignancy that can be fatal.^1^ The most important risk factors for BC are reported to be cigarette smoking, followed by petrochemical exposure.^2^ BC often has a high frequency of recurrence with unsatisfactory outcomes as the tumors progress and become more invasive.^3^ Statistics indicate that most patients diagnosed with BC usually have non-invasive tumors present.^4^ Transurethral resection is the most common treatment method for early-stage BC. Radical cystectomy is required when tumors reoccur and progress to a muscle-invasive form.^5, 6^ Limited treatment strategies are available, and approximately 31–78% of BC patients posttreatment experience a potentially lethal local recurrence within 5 years.^7, 8^ Therefore, to alter the disease course, stronger molecular parameters must be identified. This will help to monitor the progression, recurrence and metastasis of BC.^8, 9^

MicroRNAs (miRNAs) of 17–25 nucleotides in length can regulate gene expression by binding to the 3′-untranslated regions of target genes as well as have essential roles in various biological processes, including development, differentiation, cell proliferation, and apoptosis.^10, 11^ Previous studies have shown that miRNAs can be used as diagnostic and prognostic biomarkers for patients with BC.^12, 13, 14^ MicroRNA-135a (miR-135a) has been confirmed to have a role in BC.^15^ It is widely reported that miRNAs have central roles in regulating gene expression associated with carcinogenesis and tumor suppression.^16^ Accordingly, new computational approaches that identify changes in miRNA expression levels may involve the epithelial–mesenchymal transition (EMT) in several cancers, such as BC, ovarian cancer and urothelial carcinoma.^17, 18, 19^ EMT, one of the major molecular mechanisms, is important in progressing cancer during oncogenesis, cancer metastasis and drug resistance.^20, 21, 22^ It is characterized by a breakdown of cell–cell adhesion, loss of epithelial phenotypes and cell depolarization, thus accelerating cancer progression.^23^ Previous studies have indicated that many miRNAs are involved in regulating the EMT process of BC. The downregulated hsa-miR-145-5p and hsa-miR-214-3p may modulate EMT expression in patients with BC.^16^ miR-429 may reverse EMT by restoring E-cadherin expression in BC.^24^ miR-221 can also facilitate the transforming growth factor-beta1-induced EMT in human BC cells^25^ The Wnt/β-catenin signaling pathway is reported to be involved in the occurrence and progression of both EMT and cancer metastasis.^26^ A previous study highlighted glycogen synthase kinase 3β (GSK3β) as a Wnt/β-catenin signaling pathway inhibitor and provided evidence of miR-135a’s ability to promote Wnt/β-catenin signaling by suppressing GSK3β.^27^ Based on this information, we hypothesized that miR-135a may be correlated with GSK3β, EMT and the Wnt/β-catenin signaling pathway in BC. In this study, we examined the effects of miR-135a on the EMT, migration and invasion ability of BC by targeting GSK3β through the Wnt/β-catenin signaling pathway.

## Materials and methods

### Study subjects

Between September 2011 and September 2013, 165 paired BC tissues and adjacent normal tissues were obtained from patients with BC who had undergone a bladder resection confirmed by the pathology department of the Second Affiliated Hospital of Zhejiang University Medical College. The patients included 89 males and 76 females with a median age of 62 years (ranging from 28 to 84 years). Pathological grades of BC were assessed by the standards set by the World Health Organization/International Society of Urological Pathology, 2004 edition. There were 68 cases of low-grade urinary tract epithelial cancer, 46 cases of low malignant potential urothelial papillary cancer and 51 cases of high-grade urinary tract epithelial cancer. Per the standards for tumor node metastasis staging by the Union for International Cancer Control in 2002, there were 71 cases of non-muscle invasive BC in the Tis–T1 stages and 94 cases of muscle invasive BC in the T2–T4 stages. All patients participating in the study did not receive chemotherapy, radiotherapy or biological therapy prior to the study. The tissues were stored at −80 °C until further analysis. The study was performed in accordance with the guidelines established by The Medicine Ethics Review Committee of the Second Affiliated Hospital of Zhejiang University Medical College. All patients in the study signed the required consent documentation.

### Immunohistochemistry

The BC and adjacent normal tissues were fixed in 10% formaldehyde, embedded in paraffin and sectioned at a thickness of 3~4 μm. The sections were placed in 3% H_2_O_2_, dewaxed and dehydrated at room temperature for 20 min to block the endoperoxidase activity. The sections were placed in an 80% power microwave for 5 min at 90 °C for antigen retrieval. The sections were incubated overnight with mouse anti-human GSK3β monoclonal antibody (ANT-404, ProSpec-Tany Techno-Gene Ltd, Rehovot, Israel) at 4 °C and then incubated with goat anti-mouse IgG-horseradish peroxidase (HRP; SE134, Beijing Solarbio Science & Technology Co., Ltd., Beijing, China) at 37 °C for 30 min. Hematoxylin (C0105, Beyotime Biotechnology Co., Shanghai, China) was applied for 30 s to stain the nucleus, and diaminobenzidine (P0202, Beyotime Biotechnology Co.) was used for development. The sections were dehydrated until transparent using hydrochloric acid ethanol, sealed with gum, photographed and observed microscopically. The standards used for evaluating the immunohistochemical results were that if the number of stained cells exceeded 25%, and there were obvious brown or yellow–brown granules observed in the cytoplasm, the specimen was positive for GSK3β. The expression rate was determined by the percentage of positive cases from the total cases.

### Quantitative real-time PCR

Approximately 100 mg of tissue was collected, and the total RNA was extracted using a Trizol Kit (16096020, Thermo Fisher Scientific, Grand Island, NY, USA). Ten μl of RNA was diluted 20 times with RNA-free super pure water, and its optical density (OD) value was measured at 260 and 280 nm to detect the concentration and purity of the RNA. Total RNA was calculated by *V*=1.25/OD_260_ (μl). Eppendorf (EP) tubes were filled with 5 μl of mix (4368702, Tideradar Biomart, Beijing, China), 5 μl of total RNA and 10 μl of RNAse-free H_2_O. The samples were centrifuged, mixed and placed in a qPCR instrument. The reaction conditions were maintained at 37 °C for 15 min, followed by 85 °C for 5 s and termination at 4 °C. The cDNA was stored in a freezer at −20 °C. The ABI 7500 quantitative real-time PCR (qRT-PCR) instrument (ABI 7500, Applied Biosystems, Carlsbad, CA, USA) was used for the qRT-PCR assay. The reaction conditions included predenaturation at 95 °C for 10 min, denaturation at 95 °C for 10 s, annealing at 60 °C for 20 s, extension at 72 °C for 34 s for 40 cycles and followed with SYBRGreen Fluorescent dye (RR091A, Takara Holdings Inc., Kyoto, Japan). The primer sequences (Invitrogen, Shanghai, China) are listed in Table 1. U6 was used as an internal control for miR-135a, and glyceraldehyde-3-phosphate dehydrogenase (GAPDH) was used for GSK3β, β-catenin, cyclinD1, E-cadherin and vimentin. The 2^−ΔΔCt^ method was used to detect relative mRNA expression of the target gene: ΔΔCT=ΔCt_experimental group_−ΔCt_control group_, ΔCt=Ct_target gene_−Ct_internal control_. 2^−ΔΔCt^ referred to the relative transcript level of the target gene mRNA. This method was used for the subsequent cell experiments.

### Western blotting

Total protein was extracted using a Total Protein Extraction Kit (C0481, Sigma, St Louis, MO, USA). A total of 100 mg of frozen tissue was placed into preconfigured protein lysate (400 μl), placed on ice for 5 min and repeated three times. The homogenate was transferred to a 1.5 ml EP tube and centrifuged (12 000 r.p.m. × 10 min) at 4 °C. The supernatant was then transferred to an EP tube, and the concentration was measured using a BCA Kit (BCA1-1KT, Sigma).

The EP tube containing the extracted protein was added with an equal volume of 2 × sodium dodecyl sulfate-loading buffer and boiled for 5 min to denature the protein. The sample was then applied after a cooling period. The electrophoresis bath was filled with an electrophoretic buffer, and the samples were run on electrophoresis at a constant voltage of 110 V for 1–1.5 h. An appropriate size filter was cut from the transmembrane nitrocellulose. The polyvinylidene fluoride membrane was soaked in transfer buffer and installed in a ‘sandwich’ device. The membrane was transferred and run on electrophoresis (600 mA) for a 30 min period. Next the membrane was washed three times with TBST for 5 min on a shaking table and blocked in bags with a sealing fluid for 1 h. The membrane was again washed three times with TBST and added with rabbit anti-human vimentin (1:1000, SR0746), GSK3β (1:5000, SR5436), β-catenin (1:5000, CRM-006), cyclinD1 (1:10000, SR0617), E-cadherin (1:50, SR0609) and HRP-labeled rabbit anti-human GAPDH (1: 500, 10494-1-AP) (ProSpec-Tany Techno-Gene Ltd, Rehovot, Israel). The membrane was then sealed in bags and placed in the shaking table overnight at 4 °C. The membrane was washed three times with TBST for 5 min the following day. The membranes were then transferred to new bags and incubated with TNST diluted HRP-labeled goat anti-rabbit secondary antibody (1:5000, P0265, Beyotime Biotechnology Co.) for 1 h at room temperature. The samples were again washed three times using TBST for 5 min in the shaking table at room temperature, exposed, developed and analyzed. This method was used for the subsequent cell experiments.

### Dual luciferase reporter gene assay

The http://www.microRNA.org website was used to predict the target gene of miR-135a. The dual luciferase reporter gene assay was conducted to confirm whether GSK3β was the target gene of the miR-135a. The full length of the 3′-untranslated region (ATAAGAATGCGCCGCTCCTCTCCCCATTCAAC) region of amplified GSK3 gene was cloned. The PCR products were cloned into multiple cloning sites downstream of pmirGLO (E1330, Promega, Madison, WI, USA). The luciferase gene was employed to construct the pGSK3β wild type (pGSK3β-Wt). The pGSK3β mutant type (pGSK3β-Mut) was constructed based on the site-directed mutation of the miR-135a-binding sites as well as its target gene. The pRL-TK vector (E2241, Promega) expressing Renilla luciferase was used as a reference to adjust the difference in cell numbers and transfection efficiency. miR-135a mimics and the negative control (NC) were co-transfected with the luciferase reporter vector into the BC cells. The dual luciferase activity was detected per Promega’s recommended method.

### Cell culture

Normal human SVHUC-1 bladder epithelial cells (HUCL-022, iCell Bioscience Inc., Shanghai, China) and human BC cells, EJ, T24, BIU87, ScaBER and 5637 (ATCC 1089, ATCC 1057, QDC110, ZY-H792, ATCC-9532, respectively), from the American Type Culture Collection (ATCC, Manassas, VA, USA) were cultured in RPMI 1640 medium (22400089, Gibco Company, Grand Island, NY, USA) containing 10% fetal bovine serum. The cells were seeded into a six-well plate at a density of 1 × 10^5^ cells per well and incubated at 37 °C with 5% CO_2_ and saturated humidity. The medium was changed every 2–3 days. The cells were passaged if the confluence reached 80–90%. After the medium was discarded, the cells were washed twice with phosphate-buffered saline (PBS) and digested with 0.25% trypsin for 2~5 min. The cells were suspended in 5 ml of Dulbecco’s Modified Eagle’s medium (190040, Gibco Company) containing 10% fetal bovine serum and then subcultured.

### Cell grouping and transfection

The T24 cells in the logarithmic growth phase were selected and assigned to the blank group (no transfection), the NC group (transfected with empty plasmids), the miR-135a mimics group (transfected with the miR-135a mimics sequence), the miR-135a inhibitors group (transfected with the miR-135a inhibitors sequence), the siRNA-GSK3β group (transfected with the GSK3β-siRNA plasmids) or the miR-135a inhibitors+siRNA-GSK3β group (transfected with the miR-135a inhibitors sequence+GSK3β-siRNA plasmids). The sequences of the miR-135a inhibitors, miR-135a mimics (Shanghai GenePharma Co., Ltd., Shanghai, China) and the GSK3β-siRNA (Guangzhou RiboBio Co., Ltd., Guangzhou, China) are listed in Table 2. One day before the transient transfection with Lifopectamine2000, the T24 cells in the logarithmic growth phase were seeded into a 12-well plate at the concentration of 1 × 10^5^ml^−1^. When the confluence reached 50–70%, the cells were added with 800 μl serum-free medium and supplemented with the mix of miR-135a inhibitors, miR-135a mimics or GSK3β-siRNA (dissolved with Opti-MEM) and lipo 2000 (11668027, Thermo Fisher Scientific). The medium was changed after 6, and 48 h posttransfection, and the cells were observed under a fluorescence microscope. The cells were collected and the total RNA and protein were extracted for further experiments.

### Cell counting Kit-8 assay

The transfected BC cells (2 × 10^3^ml^−1^) were seeded into 96-well plates with a 100-μl medium in each well. The cells were observed at time intervals of 0, 24, 48, 72 and 96 h. The 10 μl Cell Counting Kit-8 kit (C0037, Beyotime Biotechnology Co.) was added to each well (1:10). The cells were cultured at 37 °C for 1–2 h, and then the enzyme-labeled instrument (Multiskan FC, Thermo Fisher Scientific) was applied. The OD values were measured at 450 and 630 nm. Each group had three parallel wells’ set, and the average value was calculated. The experiment was repeated three times. A cell viability curve was constructed with time as the abscissa and the OD values as the ordinates.

### Scratch test

The transfected wells (5 × 10^5^) were seeded into a six-well plate. When the confluence reached 90%, a sterilized gun head was used to scratch the well axis. The floating cells were removed with PBS, and the cells were cultured in a serum-free medium for cell recovery for 0.5–1 h. After cell recovery, the cells were photographed at 0 and 48 h. The Image-Pro Plus Analysis software (Media Cybernetics, Rockville, MD, USA) was used to measure the cell migration distance, and the average distance was calculated.

### Transwell assay

Matrigel (356234, Becton-Dickinson Pharmingen, San Diego, CA, USA) was dissolved at 4 °C overnight, diluted with serum-free medium (1:3), added to the upper chambers with 50 μl in each well and balanced in an incubator for 30 min. The cell suspensions (1 × 10^5^ml^−1^) were seeded into the upper chambers containing serum-free medium, and a medium containing 10% fetal bovine serum was added into the lower chambers. The cell invasion abilities were evaluated from the cell numbers in the Matrigel.

### Flow cytometry

After the 24-h transfection, the medium was discarded and the cells were washed with PBS. The cells were digested with 0.25% trypsin. When the cells contracted and became rounded microscopically, the digestion solution was discarded. Medium containing the serum was then added to stop digestion. The cells were dissociated into a cell suspension. The suspension was centrifuged at 1000 r.p.m. per min for 5 min, and the supernatant was removed. The cells were washed twice with PBS, fixed in precooled 70% ethanol for 30 min, centrifuged and collected. After being washed with PBS, the cells were stained with 1% propidium iodide (PI) containing RNA for 30 min. The cells were again washed twice with PBS to remove PI. The volume was adjusted to 1 ml with PBS. The cell cycle was detected by BD-Aria flow cytometry (FACSCalibur, Beckman Coulter Life Sciences, Brea, CA, USA). The experiment was repeated three times.

After 48-h transfection, the cells were digested with pancreatin without EDTA, collected in flow tubes and centrifuged. The supernatant was discarded. The cells were washed three times with cooled PBS and centrifuged. The supernatant was again discarded. Per the Annexin-V-FITC (fluorescein isothiocyanate) Cell Apoptosis Assay Kit (C1065, Beyotime Biotechnology Co.), the Annexin-V-FITC, PI and 4-(2-hydroxyerhyl) piperazine-1-erhanesulfonic acid (HEPES) (1:2:50) were prepared in Annexin-V-FITC/PI dye liquor. Every 100 μl of the dye liquor was used to re-suspend 1 × 10^6^ cells. The samples were mixed, cultured for 15 min at room temperature and then added with 1 ml HEPES. Flow cytometry was used to record the excitation wavelengths at 488 nm. Excitation was recorded at 525 nm for FITC followed by 620 nm for the PI to detect cell apoptosis.

### Tumorigenicity assay in nude mice

Thirty male specific pathogen-free-grade BALB/c nude mice (SCXK-2013, Laboratory Animal Research Center, Hubei, China), aged 4–6 weeks, weighing 15–20 g, were selected and divided into the blank group, NC group, miR-135a mimics group, miR-135a inhibitors group, siRNA-GSK3β group or miR-135a inhibitors+siRNA-GSK3β group (five mice per group). After anaesthetizing all mice with ethyl ether, the transfected cells were injected into the soft skin of the right hind leg and back at a concentration of 1 × 10^6^ cells. The mice were fed under the same conditions and observed once a day for 7 days. The length (*L*) and width (*W*) of the resulting tumors were recorded, and the tumor volume was calculated by *V*=(*L* × *W*^2^)/2. On day 35, the mice were killed and their tumors were removed and weighed. The study was approved by the Animal Ethics Committee of the Second Affiliated Hospital of Zhejiang University Medical College.

### Statistical analysis

All obtained data were analyzed using the SPSS 21.0 statistical software (SPSS Inc., Chicago, IL, USA). The data are presented as the mean±s.d. Comparisons between two groups were conducted by a *t*-test, while comparisons among multiple groups were conducted by a one-way analysis of variance. *P*<0.05 indicated a significant difference.

## Results

### Positive protein expression of GSK3β in BC tissues and their adjacent normal tissues

**Figure 1:**
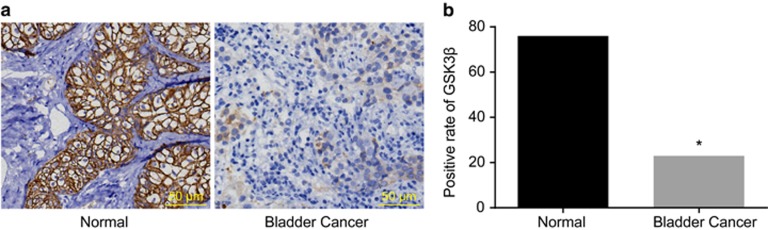
Protein expression (**a**) and positive expression rate (**b**) of GSK3β in bladder cancer tissues and their adjacent normal tissues (× 200).

Immunohistochemistry detected GSK3β expression in BC tissues and their adjacent normal tissues. The results indicated that GSK3β was mainly expressed in the cytoplasm. The positive expression rate of GSK3β in the adjacent normal tissues (46.06%) was significantly higher (*P*<0.05) than that of the BC tissues (13.94%). This indicated that GSK3β was downregulated in the BC tissues (Figure 1).

### miR-135a expression and mRNA and protein expression of GSK3β, β-catenin, cyclinD1, vimentin and E-cadherin in BC tissues and adjacent normal tissues

**Figure 2:**
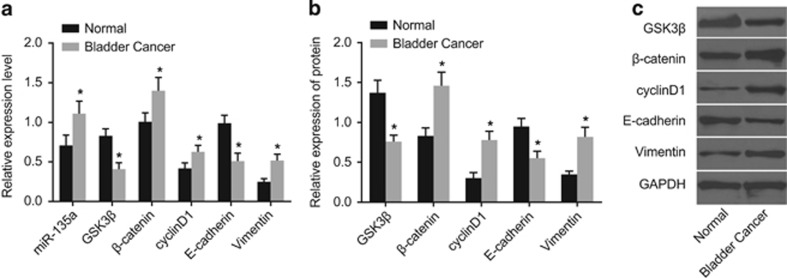
Expression levels of miR-135a expression (**a**) and expression levels of GSK3β, β-catenin, cyclinD1, vimentin and E-cadherin mRNA expression (**a**) and protein expression (**b**, **c**) in bladder cancer tissues and their adjacent normal tissues. *P<0.05 compared with adjacent normal tissues.

Western blotting and qRT-PCR were conducted to evaluate miR-135a expression as well as mRNA and protein expression of GSK3β, β-catenin, cyclinD1, vimentin and E-cadherin. The results (Figure 2) showed that the miR-135a expression and the mRNA and protein expression in β-catenin, cyclinD1 and vimentin were significantly increased compared with their adjacent normal tissues, while GSK3β and E-cadherin mRNA and protein expression were significantly decreased in BC tissues (*P*<0.05). Thus miR-135a was increased, GSK3β was decreased and the Wnt/β-catenin signaling pathway was activated in the BC tissues, which led to the EMT.

### The targeting relationship between GSK3β gene and miR-135a

**Figure 3:**
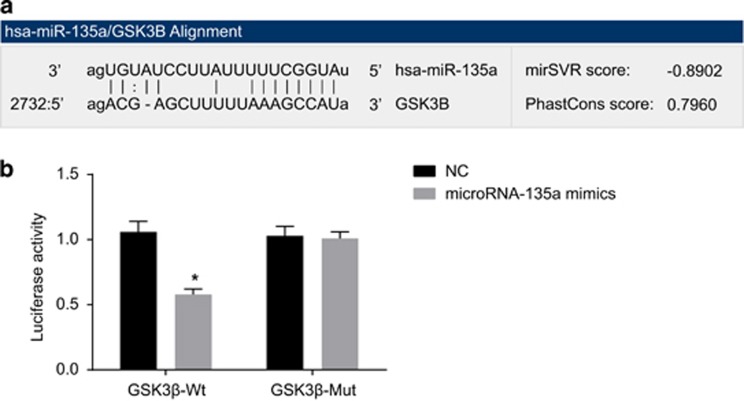
The targeting relationship between GSK3β gene and miR-135a. (**a**) The sequences of the 3′UTR region in the binding sites of GSK3β mRNA and miR-135a; (**b**) luciferase activity in the NC and miR-135a mimics groups; NC, negative control; *P<0.05 compared with the NC group; Wt, wild type; Mut, mutant.

GSK3β was only one of the putative targets of miR-135a with a lower sensitivity in comparison to the other potential targets (Figure 3a). The results of the dual luciferase reporter gene assay suggested that the luciferase signal of Wt-miR-135a/GSK3β was decreased (*P*<0.05) compared with the NC group, while that of the Mut-miR-135a/GSK3β exhibited no significant differences in the miR-135a mimics group (*P*>0.05) (Figure 3b). Therefore, miR-135a was capable of binding specifically to GSK3β.

### miR-135a expression in human normal epithelial cells (SVHUC-1) and BC cells (EJ, T24, BIU87, ScaBER and 5637)

**Figure 4:**
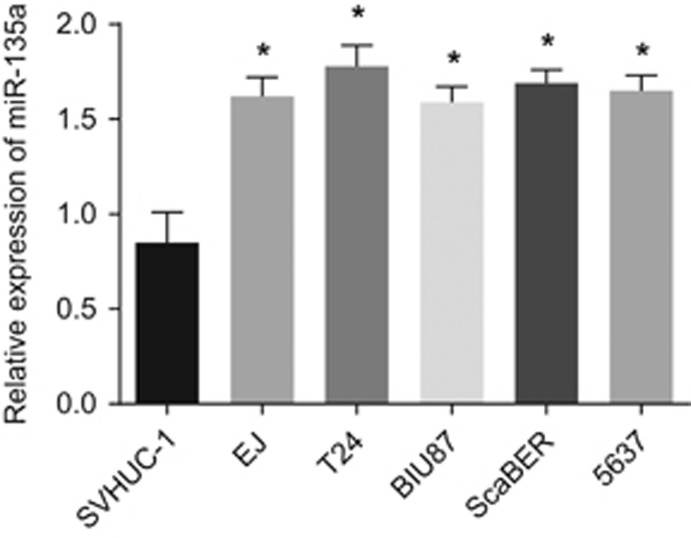
miR-135a expression in SVHUC-1 normal human epithelial cells and bladder cancer cells EJ, T24, BIU87, ScaBER and 5637. *P<0.05 compared with SVHUC-1 cell.

qRT-PCR was performed to measure miR-135a expression in normal human epithelial cells and BC cells. As shown in Figure 4, compared with SVHUC-1 normal human epithelial cells, miR-135a expression was higher in the BC cells EJ, T24, BIU87, ScaBER and 5637 (*P*<0.05). Among the five BC cell lines, miR-135a expression was the highest in the T24 cells. Thus the T24 cells were chosen for further analysis.

### miR-135a expression and mRNA and protein expression of GSK3β, β-catenin, cyclinD1, vimentin and E-cadherin among the six transfected groups

**Figure 5:**
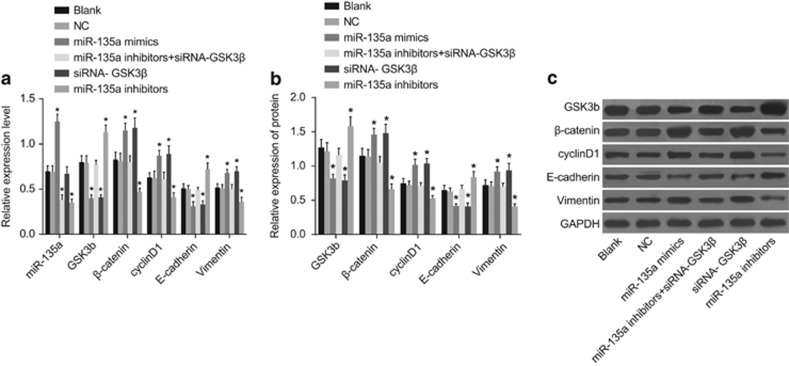
Expression of miR-135a (**a**) and mRNA (**a**) and protein (**b**, **c**) expression of GSK3β, β-catenin, cyclinD1, vimentin and E-cadherin in the six groups. (i) The blank group; (ii) the NC group; (iii) the miR-135a mimics group; (iv) the miR-135a inhibitors+siRNA-GSK3β group; (v) the siRNA-GSK3β group; (iv) the miR-135a inhibitors group. *P<0.05 compared with the blank and NC groups; NC, negative control.

Western blotting and qRT-PCR were conducted to detect miR-135a expression and mRNA and protein expression in GSK3β, β-catenin, cyclinD1, vimentin and E-cadherin. Figure 5 shows no significant differences in these factors between the blank and NC groups (*P*>0.05). However, miR-135a expression was upregulated in the miR-135a mimics group compared with the blank and NC groups, while there were no notable differences in the siRNA-GSK3β group. β-Catenin, cyclinD1 and vimentin mRNA and protein expression increased, while GSK3β and E-cadherin mRNA and protein expression decreased in the miR-135 mimics and siRNA-GSK3β groups (*P*<0.05). miR-135a expression and β-catenin, cyclinD1 and vimentin mRNA and protein expression decreased, while GSK3β and E-cadherin mRNA and protein expression increased in the miR-135a inhibitors group (*P*<0.05). miR-135a expression was significantly decreased in the miR-135a inhibitors+siRNA-GSK3β group (*P*<0.05). GSK3β, β-catenin, cyclinD1, vimentin and E-cadherin mRNA and protein expression in the miR-135a inhibitors+siRNA-GSK3β group were not significantly different compared with the blank and NC groups (*P*>0.05). These results suggest that upregulating miR-135a inhibited the GSK3β mRNA and protein expression, while promoting that of the other related genes in the Wnt/β-catenin signal pathway, resulting in EMT progression.

### Cell proliferation among the six transfected groups

**Figure 6:**
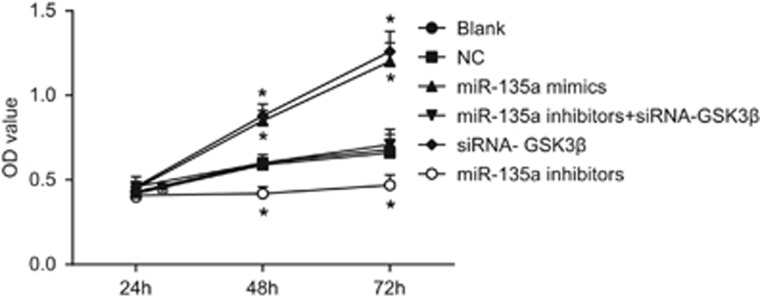
Cell growth of bladder cancer T24 cells in the six groups. *P<0.05 compared with the blank and NC groups; NC, negative control.

Compared with the blank and NC groups, T24 cells grew faster at 48 and 72 h, and the OD value was higher in the miR-135a mimics and siRNA-GSK3β groups (*P*<0.05). T24 cells grew significantly slower in the miR-135a inhibitors group (*P*<0.05). There were no significant differences in cell proliferation among the miR-135a inhibitors+siRNA-GSK3β, blank and NC groups (*P*>0.05) (Figure 6). These results suggest that overexpressed miR-135a accelerated BC cell proliferation *in vitro*, while inhibiting miR-135a also inhibited cell proliferation *in vitro*.

### Cell migration among the six transfected groups

**Figure 7:**
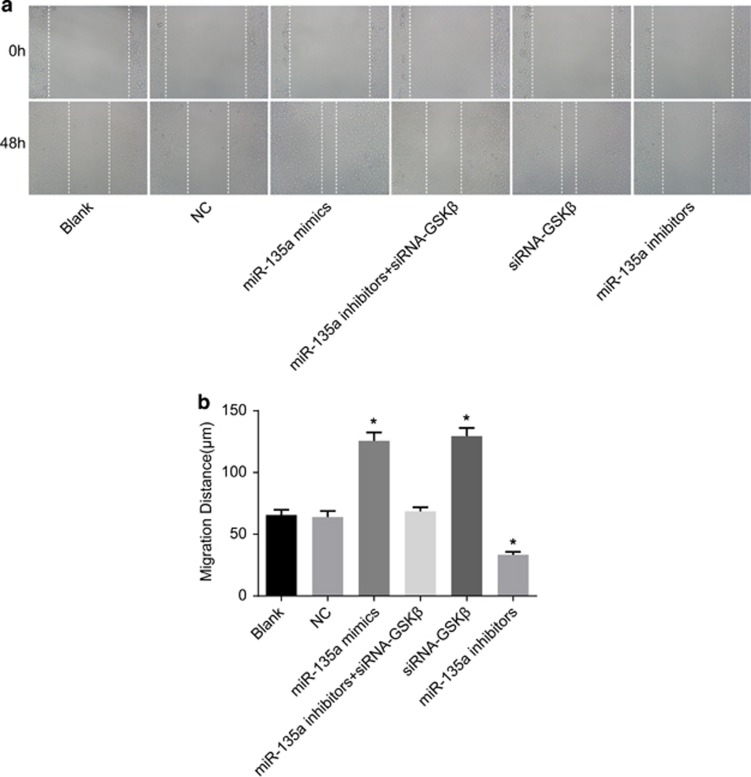
Cell migration of bladder cancer T24 cells in the six groups. (**a**) Cell migration at 0 and 48 h in the six groups; (**b**) the cell migration distance; *P<0.05 compared with the blank and NC groups; NC, negative control.

No significant differences were detected in the cell migration abilities between the blank and NC groups (*P*>0.05). Compared with the blank and NC groups, cell migration abilities increased in the miR-135a mimics and siRNA-GSK3β groups, while they decreased in the miR-135a inhibitors group (*P*<0.05). Cell migration ability in the miR-135a inhibitors+siRNA-GSK3β group was not significantly different compared with the blank and NC groups (*P*>0.05) (Figure 7). The results revealed that miR-135a overexpression enhanced BC cell migration ability *in vitro*.

### Cell invasion among the six transfected groups

**Figure 8:**
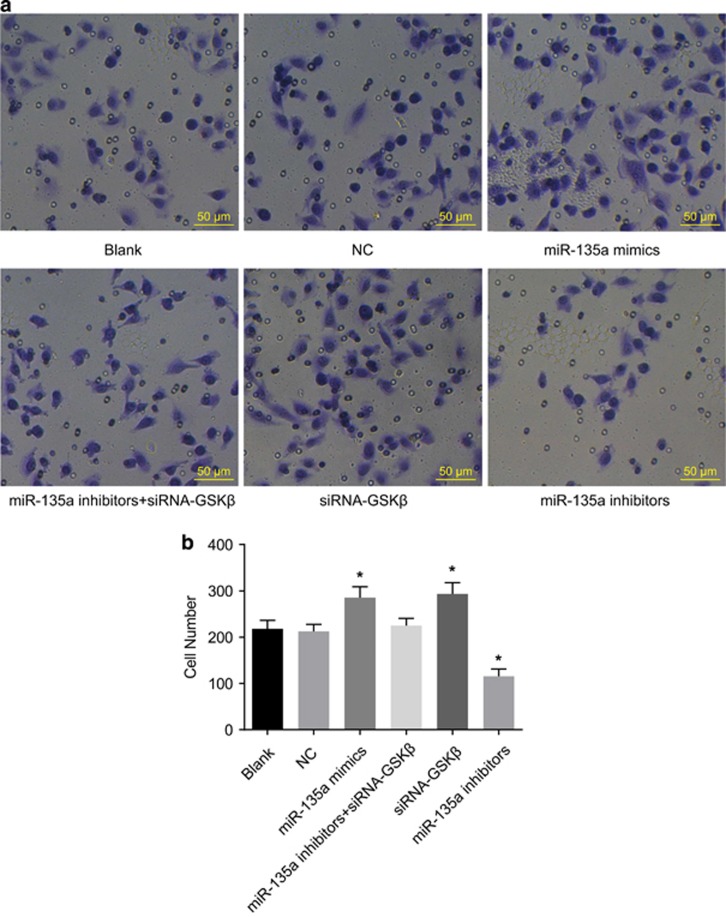
Cell invasion of bladder cancer T24 cells in the six groups (× 100). (**a**) Results of transwell assay; (**b**) cell invasion number; *P<0.05 compared with the blank and NC groups; NC, negative control.

As shown in Figure 8, there were no significant differences in cell invasion ability between the blank and NC groups (*P*>0.05). Compared with the blank and NC groups, cell invasion ability increased in the miR-135a mimics and siRNA-GSK3β groups, while it decreased in the miR-135a inhibitors group (*P*<0.05). Cell invasion ability was not significantly different in the miR-135a inhibitors+siRNA-GSK3β group compared with the blank and NC groups (*P*>0.05). This suggests that overexpression of miR-135a enhanced BC cell invasion ability *in vitro*.

### Cell apoptosis among the six transfected groups

**Figure 9:**
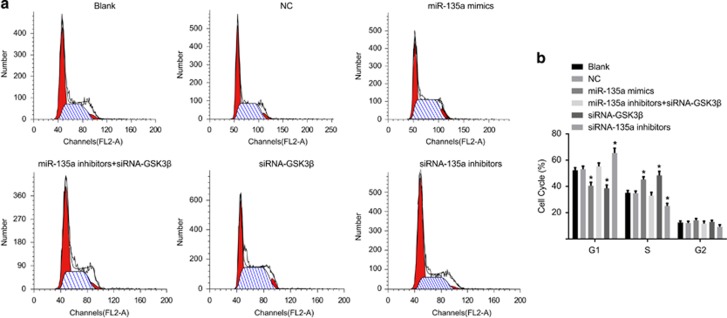
Bladder cancer T24 cell cycles at 48 h posttransfection in the six groups. (**a**) Images for cell cycle; (**b**) comparisons of cell cycle among the six groups; *P<0.05 compared with the blank and NC groups; NC, negative control.

Results of PI single staining inferred that the cellular proportions in the G1 phase in the blank, NC, miR-135a mimics, miR-135a inhibitors+siRNA-GSK3β, siRNA-GSK3β and the miR-135a inhibitors groups were 52.34±1.95%, 53.10± 2.36%, 40.52±2.56%, 55.25±2.57%, 38.56±2.57% and 65.51±3.56%, respectively. The proportions of cells in the S phase were 35.15±1.75%, 34.79±1.67%, 45.27±2.11%, 32.96±2.56%, 48.46±3.01% and 25.15±1.98%, respectively. The proportions of cells in the G2 phase were 12.51±1.21%, 12.11±1.34%, 14.21±1.35%, 11.79±1.75%, 12.98±1.21% and 9.26±1.52%, respectively. No significant differences were found in the proportions of cells in the G1 and S phases between the blank and NC groups (*P*>0.05). Compared with the blank and NC groups, G1 phase cells were decreased while S phase cells were increased in the miR-135a mimics and siRNA-GSK3β groups (*P*<0.05). Cells were stagnate in the G1 phase, and the S-phase cells were decreased in the miR-135a inhibitors group (*P*<0.05). There were no significant differences in the cell proportions in the G1 and S phases in the miR-135a inhibitors+siRNA-GSK3β group compared with blank and NC groups (*P*>0.05) (Figure 9).

**Figure 10:**
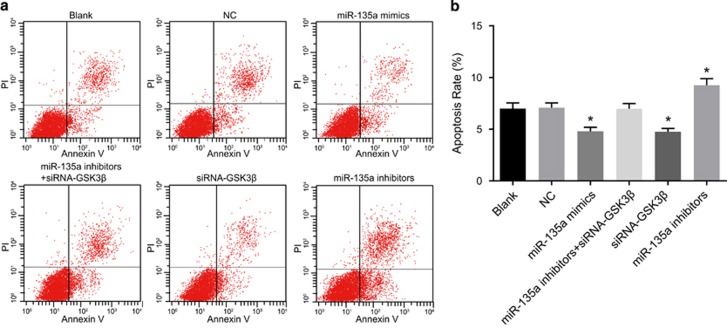
Bladder cancer T24 cell apoptosis at 48 h posttransfection in the six groups. (**a**) Images for cell apoptosis observed under a microscope; (**b**) comparisons of cell apoptosis rates among the six groups; *P<0.05 compared with the blank and NC groups; NC, negative control.

Results of Annexin V/PI indicated that the cell apoptosis rates were 7.01±0.53%, 7.08±0.47%, 4.81±0.38%, 6.98±0.51%, 4.76±0.32% and 9.25±0.64% at 48 h posttransfection in the blank, NC, miR-135a mimics, miR-135a inhibitors+siRNA-GSK3β, siRNA-GSK3β and miR-135a inhibitors groups, respectively. No significant differences were found in cell apoptosis between the blank and NC groups (*P*>0.05). Compared with the blank and NC groups, the cell apoptosis rates decreased in the miR-135a mimics and siRNA-GSK3β groups, while they increased in the miR-135a inhibitors group (*P*<0.05). Cell apoptosis rates in the miR-135a inhibitors+siRNA-GSK3β group were not significantly different compared with the blank and NC groups (*P*>0.05) (Figure 10). These results demonstrate that miR-135a overexpression inhibited BC cell apoptosis, whereas inhibiting miR-135a promoted BC cell apoptosis.

### Tumor size and growth of the mice in all groups

**Figure 11:**
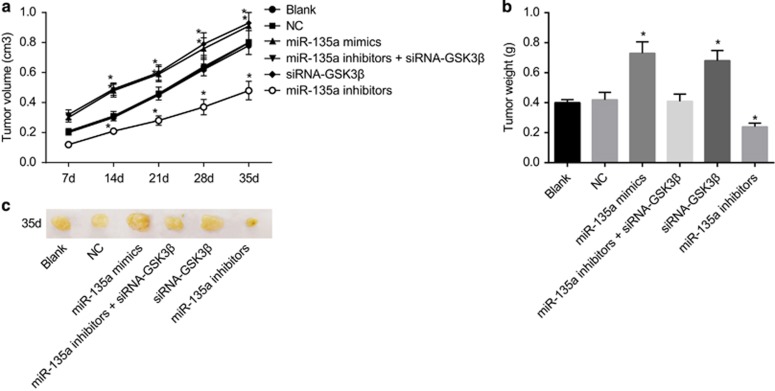
Tumor volume (**a**), tumor weight (**b**) and tumor size (**c**) of mice in the six groups. *P<0.05 compared with the blank and NC groups; NC, negative control.

Tumorigenicity assay results in the mice showed no significant differences between the blank and NC groups (*P*>0.05), and tumor size in the miR-135a mimics and siRNA-GSK3β groups was larger than that of the blank and NC groups. Tumor growth rates were the fastest, and the differences detected were statistically significant (*P*<0.05). Tumor size and growth in the miR-135a inhibitors group was lower than that of the blank and NC groups, and the differences were statistically significant (*P*<0.05). There were no statistically significant differences in the miR-135a inhibitors+siRNA-GSK3β group (*P*>0.05) (Figure 11). These results indicate that miR-135a overexpression stimulated BC tumor growth, while the inhibiting miR-135a suppressed BC tumor growth.

## Discussion

Because it is one of the most common tumors of the urogenital system, delays in diagnosing BC are strongly related to poor prognoses. Thus there is a need for swift and accurate diagnosis of BC.^28, 29^ Data continue to show the importance of miRNAs in predicting cancer survival rates.^30^ In this study, the role of miR-135a in the invasion and migration of BC was analyzed by inducing EMT and targeting GSK3β via the Wnt/β-catenin signaling pathway. Our results indicate that miR-135a activates the Wnt/β-catenin signaling pathway and promotes EMT by inhibiting GSK3β expression, thus promoting BC invasion and migration.

Our study found that, compared with their adjacent normal tissues, miR-135a expression and β-catenin, cyclinD1 and vimentin mRNA and protein expression increased, while GSK3β and E-cadherin mRNA and protein expression decreased in BC tissues. miR-135a is an oncogene that is prominent in the development of various types of cancer.^27^ Previous studies discovered that miR-135a expression is higher in cancer tissues such as hepatocellular carcinoma and colorectal cancer, facilitating cancer growth and invasion, and may serve as a biomarker.^31, 32^ Another study indicated that activating β-catenin was related to BC progression.^33^ Consistent with the results of this study, mRNA and protein expression in cyclinD1 were shown to be upregulated in urothelial carcinoma of the bladder.^34^ As important EMT-related proteins, increased levels of vimentin and decreased levels of E-cadherin expression were observed by Xiong *et al.*^35^ GSK3β as a potential tumor suppressor has also been reported to be highly expressed in invasive human urothelial carcinomas compared with noninvasive ones.^36^

Moreover, our study revealed that β-catenin, cyclinD1 and vimentin expression was elevated, while GSK3β and E-cadherin expression was decreased in the miR-135a mimics and siRNA-GSK3β groups, suggesting that miR-135a inhibited GSK3β expression and activated the Wnt/β-catenin signaling pathway. GSK3β negatively regulates and suppresses the Wnt/β-catenin signaling pathway by phosphorylating β-catenin, resulting in proteasome proteolytic degradation of β-catenin.^37^ Activating the Wnt/β-catenin signaling pathway may lead to the nuclear translocation of β-catenin.^38^ Subsequently, β-catenin leads to the transcription of its downstream genes (C-myc, cyclinD1, MMP-7 and others) by binding to the Tcf and Lef transcription factors in the nucleus.^38, 39^ Interestingly, previous studies demonstrated that GSK3β inhibits the Wnt/β-catenin signaling pathway and that miR-135a may activate the Wnt/β-catenin signaling pathway by inhibiting GSK3β.^27^ As previously established, miR-135a is involved in the pathogenesis of colorectal cancer by suppressing adenomatous polyposis coli and activating the downstream Wnt pathway.^11^ Furthermore, as shown in our study, miR-135a overexpression induces the EMT and consequently promotes cell proliferation, invasion and migration while suppressing apoptosis. EMT is a complicated process, featuring the loss of epithelial markers such as the adherence functional protein, E-cadherin, and the acquisition of mesenchymal markers, such as vimentin and fibronectin.^40^ Regarding miR-135a treatment, downregulation of E-cadherin expression and upregulation of vimentin were previously discovered, which was consistent with our study.^20^ Mao *et al.*^15^ revealed that abnormal miR-135a expression in BC cells promoted cell proliferation, cell cycle regulation and tumorigenicity, whereas suppression of miR-135a expression inhibited cell proliferation and tumorigenesis. Liu *et al.*^41^ reported that miR-135a induces hepatocellular carcinoma cell invasion and metastasis. Another study focused on miR-92 and BC and clarified that miR-92 promoted cell proliferation, invasion and activated Wnt/c-myc/MMP7 signaling by targeting GSK3β.^42^

In conclusion, our study demonstrated the role of miR-135a in regulating the EMT and the Wnt/β-catenin signaling pathway. By targeting GSK3β, miR-135a accelerates BC cell proliferation, migration and invasion. These results may be of clinical value in treating BC. More BC cell studies are required to further confirm our results, which will help provide a better understanding of the mechanisms involved in BC.

## Figures and Tables

**Table 1:** Primer sequences of PCR

*Genes*	*Sequences (5′→3′)*
miR-135a	F:AGGCCTCGCTGTTCTCTATGGC
	R:TGTCCCCGCCGTGCG
*GSK3β*	F:GGCAGCATGAAAGTTAGCAGA
	R:GGCGACCAGTTCTCCTGAATC
β-Catenin	F:TGGTGACAGGGAAGACATCA
	R:CCATAGTGAAGGCGAACTGC
CyclinD1	F:TCAAGTGTGACCCGGACTGCCT
	R:GCACGTCGGTGGGTGTGCAA
E-cadherin	F:AACGCATTGCCACATACAC
	R:GAGCACCTTCCATGACAGAC
Vimentin	F:ACAGGCTTTAGCGAGTTATT
	R:GGGCTCCTAGCGGTTTAG
GAPDH	F:GGTGAAGGTCGGAGTCAACGG
	R:CCTGGAAGATGGTGATGGGATT
U6	F:CTCGCTTCGGCAGCACA
	R:AACGCTTCACGAATTTGCGT

Abbreviations: F, forward; GAPDH, glyceraldehyde 3-phosphate dehydrogenase; R, reverse.

**Table 2:** Transfection sequences of miR-135a inhibitors, miR-135a mimics, NC and *GSK3β-*siRNA

*Items*	*Sequences*
miR-135a inhibitors	UCACAUAGGAAUAAAAAGCCAUA
miR-135a mimics	UAUGGCUUUUUAUUCCUAUGUGA
NC	UUCUCCGAACGUGUCACGUTT
*GSK3β-*siRNA	AGGAGACCACGACCTGTTAATTTT

Abbreviation: NC, negative control.

## References

[bib1] Cui X, Shen D, Kong C, Zhang Z, Zeng Y, Lin X et al. NF-kappaB suppresses apoptosis and promotes bladder cancer cell proliferation by upregulating survivin expression *in vitro* and *in vivo*. Sci Rep 2017; 7: 40723. 28139689 10.1038/srep40723 PMC5282527

[bib2] Dinney CP, McConkey DJ, Millikan RE, Wu X, Bar-Eli M, Adam L et al. Focus on bladder cancer. Cancer Cell 2004; 6: 111–116. 15324694 10.1016/j.ccr.2004.08.002

[bib3] Wang H, Li Q, Niu X, Wang G, Zheng S, Fu G et al. miR-143 inhibits bladder cancer cell proliferation and enhances their sensitivity to gemcitabine by repressing IGF-1R signaling. Oncol Lett 2017; 13: 435–440. 28123579 10.3892/ol.2016.5388 PMC5245146

[bib4] van Rhijn BW, Burger M, Lotan Y, Solsona E, Stief CG, Sylvester RJ et al. Recurrence and progression of disease in non-muscle-invasive bladder cancer: from epidemiology to treatment strategy. Eur Urol 2009; 56: 430–442. 19576682 10.1016/j.eururo.2009.06.028

[bib5] Ozer K, Horsanali MO, Gorgel SN, Ozbek E. Bladder injury secondary to obturator reflex is more common with plasmakinetic transurethral resection than monopolar transurethral resection of bladder cancer. Cent Eur J Urol 2015; 68: 284–288. 10.5173/ceju.2015.565 PMC4643702 26568867

[bib6] Knoedler J, Frank I. Organ-sparing surgery in urology: partial cystectomy. Curr Opin Urol 2015; 25: 111–115. 25581537 10.1097/MOU.0000000000000145

[bib7] Babjuk M, Oosterlinck W, Sylvester R, Kaasinen E, Bohle A, Palou-Redorta J et al. EAU guidelines on non-muscle-invasive urothelial carcinoma of the bladder. Eur Urol 2008; 54: 303–314. 18468779 10.1016/j.eururo.2008.04.051

[bib8] Matsuo T, Miyata Y, Mitsunari K, Yasuda T, Ohba K, Sakai H. Pathological significance and prognostic implications of heme oxygenase 1 expression in non-muscle-invasive bladder cancer: correlation with cell proliferation, angiogenesis, lymphangiogenesis and expression of VEGFs and COX-2. Oncol Lett 2017; 13: 275–280. 28123555 10.3892/ol.2016.5416 PMC5245111

[bib9] Xu X, Li S, Lin Y, Chen H, Hu Z, Mao Y et al. MicroRNA-124-3p inhibits cell migration and invasion in bladder cancer cells by targeting ROCK1. J Transl Med 2013; 11: 276. 24180482 10.1186/1479-5876-11-276 PMC4228407

[bib10] Yu G, Jia Z, Dou Z. miR-24-3p regulates bladder cancer cell proliferation, migration, invasion and autophagy by targeting DEDD. Oncol Rep 2017; 37: 1123–1131. 28000900 10.3892/or.2016.5326

[bib11] Nagel R, le Sage C, Diosdado B, van der Waal M, Oude Vrielink JA, Bolijn A et al. Regulation of the adenomatous polyposis coli gene by the miR-135 family in colorectal cancer. Cancer Res 2008; 68: 5795–5802. 18632633 10.1158/0008-5472.CAN-08-0951

[bib12] Mlcochova H, Hezova R, Stanik M, Slaby O. Urine microRNAs as potential noninvasive biomarkers in urologic cancers. Urol Oncol 2014; 32: 41 e41–41 e49. 10.1016/j.urolonc.2013.04.011 24035473

[bib13] Rosenberg E, Baniel J, Spector Y, Faerman A, Meiri E, Aharonov R et al. Predicting progression of bladder urothelial carcinoma using microRNA expression. BJU Int 2013; 112: 1027–1034. 23387295 10.1111/j.1464-410X.2012.11748.x

[bib14] Wang S, Xue S, Dai Y, Yang J, Chen Z, Fang X et al. Reduced expression of microRNA-100 confers unfavorable prognosis in patients with bladder cancer. Diagn Pathol 2012; 7: 159. 23173870 10.1186/1746-1596-7-159 PMC3539897

[bib15] Mao XP, Zhang LS, Huang B, Zhou SY, Liao J, Chen LW et al. Mir-135a enhances cellular proliferation through post-transcriptionally regulating PHLPP2 and FOXO1 in human bladder cancer. J Transl Med 2015; 13: 86. 25888950 10.1186/s12967-015-0438-8 PMC4367980

[bib16] Falzone L, Candido S, Salemi R, Basile MS, Scalisi A, McCubrey JA et al. Computational identification of microRNAs associated to both epithelial to mesenchymal transition and NGAL/MMP-9 pathways in bladder cancer. Oncotarget 2016; 7: 72758–72766. 27602581 10.18632/oncotarget.11805 PMC5341942

[bib17] Braicu C, Cojocneanu-Petric R, Chira S, Truta A, Floares A, Petrut B et al. Clinical and pathological implications of miRNA in bladder cancer. Int J Nanomed 2015; 10: 791–800. 10.2147/IJN.S72904 PMC4309789 25653521

[bib18] Bagnoli M, De Cecco L, Granata A, Nicoletti R, Marchesi E, Alberti P et al. Identification of a chrXq27.3 microRNA cluster associated with early relapse in advanced stage ovarian cancer patients. Oncotarget 2015; 6: 9643. 26002433 10.18632/oncotarget.3998 PMC4496384

[bib19] Michailidi C, Hayashi M, Datta S, Sen T, Zenner K, Oladeru O et al. Involvement of epigenetics and EMT-related miRNA in arsenic-induced neoplastic transformation and their potential clinical use. Cancer Prev Res (Phila) 2015; 8: 208–221. 25586904 10.1158/1940-6207.CAPR-14-0251 PMC4355280

[bib20] Shi H, Ji Y, Zhang D, Liu Y, Fang P. MiR-135a inhibits migration and invasion and regulates EMT-related marker genes by targeting KLF8 in lung cancer cells. Biochem Biophys Res Commun 2015; 465: 125–130. 26235874 10.1016/j.bbrc.2015.07.145

[bib21] Singh A, Settleman J. EMT, cancer stem cells and drug resistance: an emerging axis of evil in the war on cancer. Oncogene 2010; 29: 4741–4751. 20531305 10.1038/onc.2010.215 PMC3176718

[bib22] De Craene B, Berx G. Regulatory networks defining EMT during cancer initiation and progression. Nat Rev Cancer 2013; 13: 97–110. 23344542 10.1038/nrc3447

[bib23] Thiery JP, Acloque H, Huang RY, Nieto MA. Epithelial-mesenchymal transitions in development and disease. Cell 2009; 139: 871–890. 19945376 10.1016/j.cell.2009.11.007

[bib24] Wu CL, Ho JY, Chou SC, Yu DS. MiR-429 reverses epithelial-mesenchymal transition by restoring E-cadherin expression in bladder cancer. Oncotarget 2016; 7: 26593–26603. 27058893 10.18632/oncotarget.8557 PMC5042001

[bib25] Liu J, Cao J, Zhao X. miR-221 facilitates the TGFbeta1-induced epithelial-mesenchymal transition in human bladder cancer cells by targeting STMN1. BMC Urol 2015; 15: 36. 25928257 10.1186/s12894-015-0028-3 PMC4423111

[bib26] Cai J, Guan H, Fang L, Yang Y, Zhu X, Yuan J et al. MicroRNA-374a activates Wnt/beta-catenin signaling to promote breast cancer metastasis. J Clin Invest 2013; 123: 566–579. 23321667 10.1172/JCI65871 PMC3561816

[bib27] Yang X, Wang X, Nie F, Liu T, Yu X, Wang H et al. miR-135 family members mediate podocyte injury through the activation of Wnt/beta-catenin signaling. Int J Mol Med 2015; 36: 669–677. 26134897 10.3892/ijmm.2015.2259 PMC4533775

[bib28] Chen F, Chen X, Yang D, Che X, Wang J, Li X et al. Isoquercitrin inhibits bladder cancer progression *in vivo* and *in vitro* by regulating the PI3K/Akt and PKC signaling pathways. Oncol Rep 2016; 36: 165–172. 27177091 10.3892/or.2016.4794

[bib29] Sun M, Trinh QD. Diagnosis and staging of bladder cancer. Hematol Oncol Clin North Am 2015; 29: 205–218 vii. 25836929 10.1016/j.hoc.2014.10.013

[bib30] Zhou H, Tang K, Xiao H, Zeng J, Guan W, Guo X et al. A panel of eight-miRNA signature as a potential biomarker for predicting survival in bladder cancer. J Exp Clin Cancer Res 2015; 34: 53. 25991007 10.1186/s13046-015-0167-0 PMC4508815

[bib31] Zhou W, Li X, Liu F, Xiao Z, He M, Shen S et al. MiR-135a promotes growth and invasion of colorectal cancer via metastasis suppressor 1 *in vitro*. Acta Biochim Biophys Sin (Shanghai) 2012; 44: 838–846. 23017832 10.1093/abbs/gms071

[bib32] von Felden J, Heim D, Schulze K, Krech T, Ewald F, Nashan B et al. High expression of micro RNA-135A in hepatocellular carcinoma is associated with recurrence within 12 months after resection. BMC Cancer 2017; 17: 60. 28100188 10.1186/s12885-017-3053-7 PMC5242004

[bib33] Li Y, Zheng Y, Izumi K, Ishiguro H, Ye B, Li F et al. Androgen activates beta-catenin signaling in bladder cancer cells. Endocr Relat Cancer 2013; 20: 293–304. 23447569 10.1530/ERC-12-0328

[bib34] Kopparapu PK, Boorjian SA, Robinson BD, Downes M, Gudas LJ, Mongan NP et al. Expression of cyclin d1 and its association with disease characteristics in bladder cancer. Anticancer Res 2013; 33: 5235–5242. 24324055 PMC4122540

[bib35] Xiong D, Liou Y, Shu J, Li D, Zhang L, Chen J. Down-regulating ribonuclease inhibitor enhances metastasis of bladder cancer cells through regulating epithelial-mesenchymal transition and ILK signaling pathway. Exp Mol Pathol 2014; 96: 411–421. 24768914 10.1016/j.yexmp.2014.04.012

[bib36] Naito S, Bilim V, Yuuki K, Ugolkov A, Motoyama T, Nagaoka A et al. Glycogen synthase kinase-3beta: a prognostic marker and a potential therapeutic target in human bladder cancer. Clin Cancer Res 2010; 16: 5124–5132. 20889919 10.1158/1078-0432.CCR-10-0275

[bib37] Guan H, Liu C, Fang F, Huang Y, Tao T, Ling Z et al. MicroRNA-744 promotes prostate cancer progression through aberrantly activating Wnt/beta-catenin signaling. Oncotarget 2017; 8: 14693–14707. 28107193 10.18632/oncotarget.14711 PMC5362436

[bib38] Clevers H. Wnt/beta-catenin signaling in development and disease. Cell 2006; 127: 469–480. 17081971 10.1016/j.cell.2006.10.018

[bib39] Anastas JN, Moon RT. WNT signalling pathways as therapeutic targets in cancer. Nat Rev Cancer 2013; 13: 11–26. 23258168 10.1038/nrc3419

[bib40] Theys J, Jutten B, Habets R, Paesmans K, Groot AJ, Lambin P et al. E-Cadherin loss associated with EMT promotes radioresistance in human tumor cells. Radiother Oncol 2011; 99: 392–397. 21680037 10.1016/j.radonc.2011.05.044 PMC4948667

[bib41] Liu S, Guo W, Shi J, Li N, Yu X, Xue J et al. MicroRNA-135a contributes to the development of portal vein tumor thrombus by promoting metastasis in hepatocellular carcinoma. J Hepatol 2012; 56: 389–396. 21888875 10.1016/j.jhep.2011.08.008

[bib42] Wang H, Ke C, Ma X, Zhao Q, Yang M, Zhang W et al. MicroRNA-92 promotes invasion and chemoresistance by targeting GSK3beta and activating Wnt signaling in bladder cancer cells. Tumour Biol 2016. DOI:10.1007/s13277-016-5460-9. 10.1007/s13277-016-5460-9 27830467

